# Student Dropout as a Never-Ending Evergreen Phenomenon of Online Distance Education

**DOI:** 10.3390/ejihpe13050069

**Published:** 2023-05-19

**Authors:** Sevgi Elibol, Aras Bozkurt

**Affiliations:** Distance Education Department, Open Education Faculty, Anadolu University, Eskisehir 26470, Turkey

**Keywords:** dropout, attrition, course retention, distance education, online learning

## Abstract

The research on student dropout demonstrates that there is no consensus on its definition and scope. Although there is an expanding collection of research on the topic, student dropout remains a significant issue, characterized by numerous uncertainties and ambiguous aspects. The primary aim of this investigation is to assess the research trends of student dropout within the distance education literature by employing data mining and analytic approaches. To identify these patterns, a total of 164 publications were examined by applying text mining and social network analysis. The study revealed some intriguing facts, such as the misinterpretation of the term “dropout” in different settings and the inadequacy of nonhuman analytics to explain the phenomenon, and promising implications on how to lessen dropout rates in open and distance learning environments. Based on the findings of the study, this article proposes possible directions for future research, including the need to provide a precise definition of the term “dropout” in the context of distance learning, to develop ethical principles, policies, and frameworks for the use of algorithmic approaches to predict student dropout, and finally, to adopt a human-centered approach aimed at fostering learners’ motivation, satisfaction, and independence to reduce the rate of dropout in distance education.

## 1. Introduction

Education has always been viewed as the key to the growth, development, and well-being of a society, and it has become even more of a prerequisite for citizens since the world is continuously evolving and the global challenges are getting more and more difficult to handle in everyday life. Recent advances in technology have made continued education and lifelong learning easier by allowing institutions to reach millions of students in a quicker and more resourceful way. This notion is also articulated in the Sustainable Development Goals, Number 4: Quality Education to provide “quality education for all is fundamental to creating a peaceful and prosperous world” [1]. Consequently, educational institutions globally have begun providing distance education courses to address this demand. Distance education has revolutionized learning by filling the geographical gap between institutions and learners [2] and, thanks to its flexible nature, offered people who have job or family commitments the opportunity to achieve their educational goals since its emergence [3].

However, despite the growing demand for distance learning, institutions suffer low retention rates within their distance learning environments [4], and it is not uncommon for learners to drop out, whether willingly or unwillingly, from these programs at some point. Furthermore, there is no single agreed-upon definition of the term “dropout”. For instance, Tinto [5] defines “dropout” as any person leaving their institution, while Kaplan et al. [6] state that dropouts are those who leave their departments voluntarily after the payment of the tuition fee is completed and/or the add/drop period is over. Conversely, Levy [7] presents a differing perspective, positing that students who choose to withdraw from a course within the “add/drop period” should not be classified as dropout students. This is due to the fact that they either receive a full refund for their tuition or do not face any financial repercussions for discontinuing the course during this specific time.

Although dropouts pose a major concern for all types of education, the dropout rate in distance education is much higher than that in traditional education, as Moore and Kearsley [8] have made clear. This, however, comes as no surprise when the spatial, temporal, and transactional distances separating the common target learner populations of distance education are considered. Providing educational opportunities to a relatively higher number of learners from any social, academic, or economic background, then, can become a downside, as high rates of dropout can be linked to a variety of issues in such a diverse context, which might prevent institutions from fully understanding and addressing the causes of dropout. Within the above-mentioned context, the overall purpose of this study is to examine the research trends and patterns of student dropout in distance education systems and to identify the emerging thematic research patterns.

Despite student dropout being a central concern for educational stakeholders since the inception of formal education systems [9], it was not until the early 1970s that theoretical frameworks began to emerge. The initial research aimed at understanding student dropout primarily centered on students’ physiological characteristics, including aspects such as personality, capabilities, and motivation [9], as well as personal deficiencies [9,10,11]. These investigations, categorized as psychological studies [9,12], were succeeded by additional theoretical models and research efforts that sought to elucidate the phenomenon from various angles, including psychological, environmental, sociological, economic, and organizational perspectives [9,10].

Since 1970, the phenomenon has been widely investigated, and various conceptual and theoretical models [5,11,13,14,15,16,17,18] have been put forward to explain the issue. These models aimed to help institutions identify at-risk students and present some implications for early interventions that practitioners and curriculum designers should be mindful of. Except for these models, there have been many other preliminary studies, most of which focus primarily on factors affecting student dropout.

In an attempt to reveal what has been said about the issue at a glance and provide a robust interpretation of all the relevant empirical evidence, there have also been several literature review studies. The first systematic review of dropout studies in the context of online learning was conducted by Lee and Choi [19]. In their seminal study, Lee and Choi [19] conducted a comprehensive analysis of 35 empirical research articles, exclusively from peer-reviewed academic publications, spanning the decade between 2000 and 2010. In order to find related studies, Lee and Choi [19] both searched for the keywords that might relate to relevant studies in three major educational databases: Education Research Complete, ERIC, and PsycINFO, and employed a snowball method. In their investigation, Lee and Choi [19] utilized the research classification frameworks established by Creswell, [20] and initially presented the authors, publication years, study samples, contexts, and dropout definitions pertaining to each individual study. Later Lee and Choi [19] focused on the factors affecting dropout and explained the phenomenon under categories such as student factors (academic background, relevant experiences, skills, and psychological attributes), course/program factors (course design, institutional support and interactions), and environment factors (work commitments and supportive environments).

Hart [21] presented a pioneering literature review that examined the factors potentially influencing students’ persistence in online courses. To select the relevant articles, Hart [21] employed the following criteria: (a) publication after 1999, (b) appearance in a peer-reviewed journal, and (c) the discussion of student factors promoting persistence. After evaluating 11 papers, Hart [21] offered a deeper understanding of persistence as a phenomenon, as well as identifying factors that encourage persistence and obstacles that hinder it.

In a more contemporary examination of the subject, Muljana and Luo [22] conducted a comprehensive review of the existing literature. Their investigation involved an analysis of 40 empirical studies published between 2010 and 2018, utilizing extensive database searches, abstract screenings, full-text evaluations, and synthesis procedures. Acknowledging the multifaceted and intricate nature of student dropout, Muljana and Luo [22] identified a plethora of contributing factors, such as institutional support, program difficulty, fostering a sense of belonging, enhancing learning, course structure, student behavior traits, demographic aspects, and other personal variables. Additionally, they found that prior academic performance, age, gender, personal circumstances, and abilities in online and self-directed learning emerged as the most prominent determinants of online dropout [22].

Pant et al. [23] were among those who aimed to provide a comprehensive picture of the literature on student dropout in online learning environments. However, Pant et al. [23] examined studies that focused on persistence specifically in MOOCs and were published between the years 2011 and 2020. In their analysis, Pant et al. [23] selected 50 research papers that investigated factors increasing student motivation and thus contributing to their persistence in MOOCs. The findings of this study demonstrated that learner-related factors, classified as personal, social, educational, and industrial, played a vital role in determining student enrollment in MOOCs.

Although not exactly the focus of this study, dropout has become more evident as an important problem in higher education during the COVID-19 pandemic. During the emergency remote teaching and learning practices implemented during the pandemic [24,25], it was observed that learners chose to drop out due to psychological, pedagogical, and socio-economic reasons. Among these reasons, psychological issues such as engagement and motivation were salient [26,27].

As can be seen in the literature, there have been many attempts to investigate students’ dropping out from online learning environments. Some of these studies were systematic reviews and contributed greatly to the existing literature since they delivered a rigorous summary and comparison of several primary studies and, therefore, addressed research questions better than a single study might do. Nevertheless, a key problem with much of the literature on the phenomenon is that none of the studies that tend to review the past literature have applied data mining and analytics methods. Employing such methods would help a study identify patterns and relationships in large volumes of data extracted from a great number of sources and represent them in a more meaningful way.

When conducting research in any discipline, it is important to identify the gaps and trends, determine the scholarly ground, and act accordingly. As reported in the literature review section, the existing research indicates that the concept of dropout in distance education is extensive, multifaceted, and multidimensional. Consequently, it is vital to comprehend the dropout phenomenon in distance education by exploring what has been previously discussed in order to gain deeper insights into potential new perspectives. In light of these considerations, the objective of the current study is to scrutinize dropout trends in the realm of distance education research. To achieve this, the study aims to address the subsequent research inquiry:

- What are the thematic research patterns for dropout studies in the field of distance education?

## 2. Materials and Methods

### 2.1. Research Design

This study employed data mining and analytics approaches [28] to examine the articles on dropout from the perspective of distance education. In addition to descriptive statistics, tSNE analysis [29], social network analysis (SNA) [30], and text mining approaches [31] were used. Titles of the articles were analyzed using t-distributed stochastic neighbor embedding (t-SNE) to visualize “high-dimensional data by giving each datapoint [words in titles and abstracts] a location in a two [sic] or three-dimensional map” [29]. SNA [30] of the keywords was performed to better identify thematic clusters and significant nodes with strategic positions in the keyword network. In this analysis, each keyword identified by the authors of the article was considered a node, and its co-occurrences were considered relationships. By adopting text mining [31] through the lexical analysis of the titles and abstracts, the researchers were able to visualize a thematic concept map and identify major themes emerging from the research corpus. By applying different data mining and analytics approaches, the data could be triangulated, thereby increasing the validity and reliability of the study [32].

### 2.2. Inclusion Criteria and Sampling

In order to obtain a broad view, Scopus, which is the largest scholarly database, was selected for the sampling of the articles and proceedings. The research corpus was created by sampling publications that met the following inclusion criteria: (1) indexed in the Scopus database, (2) written in English or translated into English, and (3) presence of search query items in the title of the articles and proceedings (Table 1). The search yielded a total of 164 publications (80 articles and 84 proceedings).

**Figure 1:**
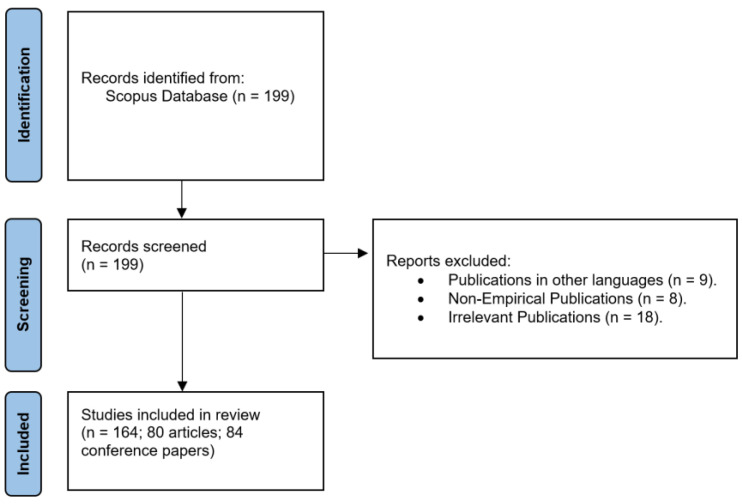
The PRISMA Protocol.

Throughout the sampling and research corpus creation processes, this study employed the PRISMA protocol, which pertains to the preferred reporting items for systematic reviews and meta-analyses (Figure 1) [33]. Ultimately, a total of 164 publications were encompassed in the final phase.

### 2.3. Data Collection Tools and Data Analysis Procedures

The data were crawled from the Scopus database, analyzed, and visualized by applying different data visualization tools. The study included a total of four phases. In the first phase of the study, time trends of the sampled studies were identified using descriptive statistics. In the second phase, the patterns in the titles were visualized by applying tSNE analysis [29]. In the third phase, the abstracts of the studies were analyzed using SNA [30] and text mining approaches [31], followed by identification of patterns and visualization of these patterns on a thematic concept map. In the final phase, using SNA [30], the keywords were analyzed based on their centrality metrics and then visualized on a network graphic.

### 2.4. Strengths and Limitations of the Study

The main strength of this study is its ability to use different data mining analytics approaches, which allowed for the visualization of the findings and identification of the research trends and patterns in an easily observable manner to facilitate analysis of the large volume of textual data. On the other hand, the study also has some limitations. First, the study examines publications that are only indexed in the Scopus database. However, publications not indexed in Scopus could have provided further insights into the research in question. Second, the study only examines peer-reviewed articles and proceedings, under the assumption that these provide the most robust and reliable findings. However, different types of publications (e.g., books, book chapters, reports, etc.) could have provided invaluable complementary findings. Third, while data mining techniques are considered a strength, they also have some limitations, and we acknowledge that our findings can only provide a partial view.

## 3. Results

In the subsequent section, the findings from various analyses are presented, including time trend examination, tSNE evaluation of the titles, text mining of abstracts, and social network analysis (SNA) of the keywords.

### 3.1. Time Trend

**Figure 2:**
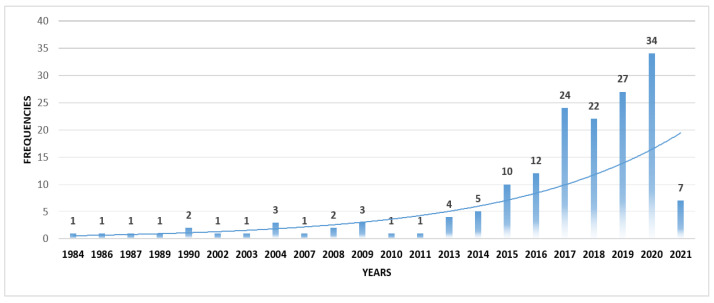
Time trends of the publications on dropout in distance education.

When the time trends of the publications are examined, it can be seen that, while there is a slow but steady increase by the first decade of the 2000s, the number of the publications increases from 2010 onward and reaches its peak by 2020 (Figure 2).

### 3.2. tSNE Analysis of the Titles

**Figure 3:**
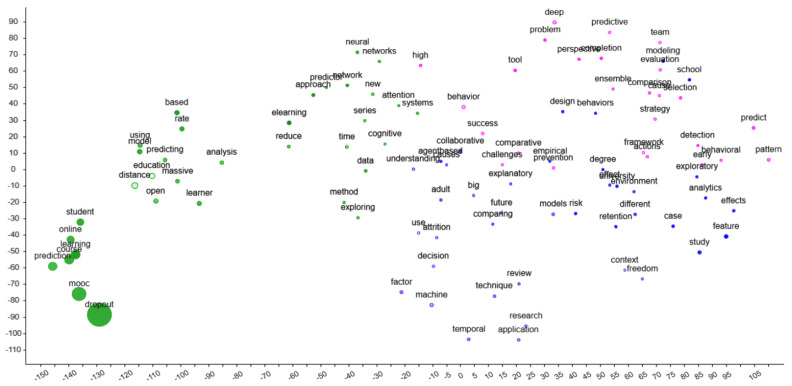
tSNE analysis of the titles in the publications on dropout in distance education.

To identify the general focal point of the publications on dropout in distance education, a tSNE analysis, an unsupervised “nonlinear dimensionality reduction technique that aims to preserve the local structure of data” [29], was conducted. The analysis indicated that the publications distinctly focused on dropout in MOOCs (see the lower left corner in Figure 3), with a specific emphasis on predicting dropouts using different machine-based statistical models. The initial findings that emerged in tSNE analysis align with the insights gained from the time trends analysis.

### 3.3. Text Mining of the Abstracts and SNA of the Keywords

**Figure 4:**
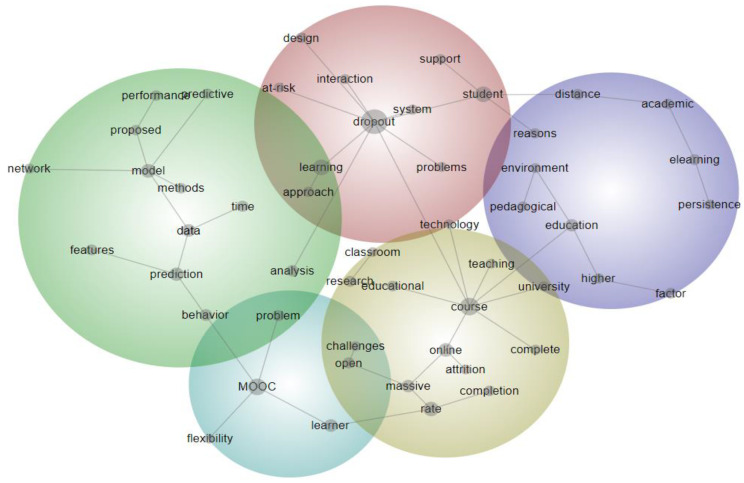
Thematic concept map of the abstracts in the publications on dropout in distance education.

**Figure 5:**
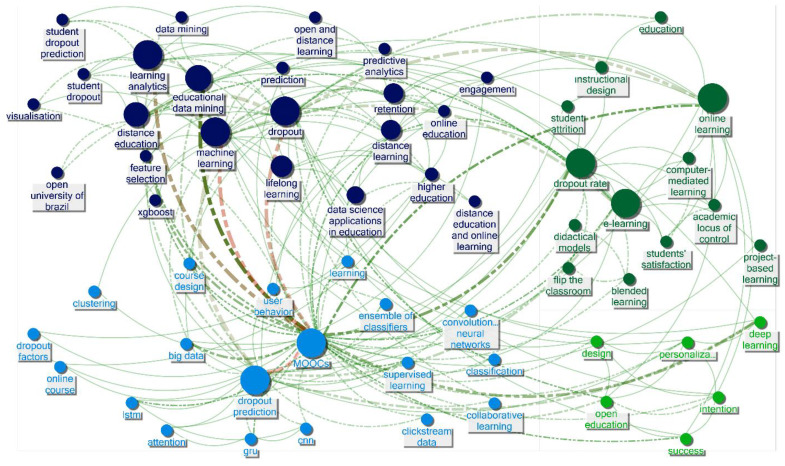
Network graph of the keywords in the publications on dropout in distance education.

In order to discern the research trends within the 164 publications addressing dropout in distance education, text mining techniques were employed to reveal patterns in the abstracts (see Figure 4), and SNA was utilized to uncover patterns in the keywords (see Figure 5). As a result, three overarching themes were identified through the triangulation of text mining and SNA findings. In this section, each theme was presented with evidence from text mining and SNA, and the entitled themes manifested themselves. Following that, we have explained these themes and discussed them in the next section by comparing and contrasting the related literature.

Theme 1: On defining dropout in MOOCs (see path in Figure 4: flexibility, MOOC, problem, completion, rate, massive open challenges, attrition, online, and massive; see nodes in Figure 5: lifelong learning, dropout rates, and MOOCs).

Theme 2: Non-human analytical data mining approaches to predict dropout (see path in Figure 4: learner, MOOC, behavior, prediction, data, model, and methods; see nodes in Figure 5: distance education, open and distance learning, dropout, learning analytics, educational data mining, machine learning, predictive analytics, data mining, and student dropout prediction).

Theme 3: Interaction, satisfaction, engagement, and personalization to reduce dropout rates (see path in Figure 4: design, interaction, dropout, learning and support, student, distance, academic, e-learning, persistence and approach, learning, dropout, and at-risk; see nodes in Figure 5: success, personalization, intention, design, academic locus of control, students satisfaction, e-learning, online learning, instructional design, students attrition, engagement, online education, distance learning, and higher education).

## 4. Discussion

### 4.1. Time Trend

The first paper in the research corpus, written by Thompson [34] and published in 1984, addressed dropout in distance education from the perspective of the cognitive style of field dependence. Other earlier papers also used an exploratory research design. For instance, Sweet [35] examined dropout to validate Tinto’s model [5] for adult distance education students. The third paper in the research corpus, which was written by Garrison [36], noted that the field needs to go beyond simply descriptive studies and focus more on comprehensive and advanced research to better understand dropout in distance education. The first publications were explorative, and they highlighted the significance of the dropout phenomenon in distance education. In the 1990s, research on dropout began to attract some attention [37], which continued at a slow but steady pace until 2010. After 2013, there was a pronounced increase in the number of studies on dropout, and by 2017, the amount of research on dropout in distance education doubled before reaching its peak in 2020. Examination of the research corpus further showed that following the emergence of the first generation cMOOCs, the second generation of xMOOCs gained a lot of popularity [38], with the high dropout rates in MOOCs turning the spotlight on MOOC research [39]. For instance, publications on MOOCs and dropout [40,41] that focused on predictions using educational data mining [42,43,44] and publications that specifically focused on dropouts in online distance education [45,46] drew much attention. The findings indicate that the emergence of MOOCs and their popularity along with the emergence of online distance learning as a frequently used model in mainstream education further triggered the research on dropouts from the perspective of distance education. Another interesting finding is that most of the publications focused on predicting dropout behavior using educational data mining.

### 4.2. Research Patterns

This section provides a discussion of the research patterns. More specifically, it identifies the research themes through text mining of the abstracts and SNA of the keywords.

#### 4.2.1. On Defining Dropout in MOOCs

A major flaw in the studies conducted to explore the reasons for students’ dropout from online environments is the lack of a proper definition of the term dropout. Previous attempts to come up with a precise definition have fallen short in many cases. In their comprehensive review, Lee and Choi [19] noted that many of the studies examined provided no general agreement on the definition of dropout from online courses, which made it quite challenging to compare dropout factors across different learning environments. Thus far, dropout students have been characterized in a variety of ways, including those who did the following:

- Departed from their institution for some reason [5];
- Voluntarily left their departments after finalizing tuition fee payments and the conclusion of the drop/add period [6];
- Did not register following three consecutive terms of non-enrollment [47];
- Earned a grade of F or formally withdrew from the course [48];
- Enrolled in a minimum of one module but failed to submit a single project [49];
- Were unable to complete a course during a semester [50];
- Went through the official withdrawal procedure [51];
- Opted to withdraw from e-learning, incurring financial penalties [7];
- Either withdrew or were dismissed from the program [52];
- Failed to meet the program requirement of completing two courses per year [53].

As for MOOCs, which globally offer open and flexible learning experiences for a large body of learners [54], the challenge to answer the question of whether every dropout is an actual dropout becomes more prominent [55]. According to Zheng et al. [56], some MOOC learners may only be interested in understanding particular concepts or some content rather than passing exams or achieving certificates, which may result in their leaving after acquiring the corresponding knowledge. Hence, previous attempts to define dropout seem to be inadequate and inconsistent due to the variety of ways that students join and leave MOOCs [57]. Furthermore, as Astin [58] stated, drawing a sharp distinction between dropouts and non-dropouts could be problematic in many ways because as long as the students in question are alive, they may return to college.

#### 4.2.2. Non-Human Analytical Data Mining Approaches to Predict Dropout

As MOOCs have grown to include a massive number of students, effective machine learning models of complex student behavior patterns that identify at-risk students are needed [59]. The benefits of a diagnosis at an early stage include the ability to provide immediate intervention for at-risk students and determine in advance whether at-risk students lack interest in the teacher, the course, or MOOC itself [60]. Existing studies have placed great emphasis on supervised learning algorithms to build discriminative models that are capable of predicting dropout [61,62]. As Henrie et al. [63] suggested, the log data of learning behaviors collected by MOOC platforms where viewing is the basic learning behavior have been used to analyze dropout rates. Nonetheless, research predominantly utilizing clickstream data to construct a flow network model of collective attention in order to examine dropout learning patterns prompts numerous inquiries concerning the sufficiency of non-human analytics for exclusively explaining this phenomenon.

The literature reveals that the reasons for student dropout may vary. Previous studies explaining dropout reasons on personal grounds have highlighted the significance of psychological attributes, such as motivation [52,64,65,66,67], self-efficacy [52,67], and satisfaction [7,48]. Moreover, Perry et al. [53] point out that unexpected life events, such as health problems encountered by the student or a family member, death of a family member, or unplanned financial pressures, may also lead to dropout. In such unanticipated circumstances, misleading or inadequate data can emerge when learning algorithms rely too heavily on learner logs.

#### 4.2.3. Interaction, Satisfaction, Engagement, and Personalization to Reduce Dropout Rates

The results emphatically indicate the need for implementing strategies to minimize learning obstacles and tackle the potential causes for withdrawal. The creation of supportive environments and provision of encouragement are deemed essential factors [4], irrespective of their origin. Support from various sources, such as family or friends [18,21,52,65,67] or the institution itself [64,68], considerably impacts students’ perseverance. Another crucial element is course satisfaction, as evidenced by multiple studies [7,48]. The level of satisfaction derived from e-learning plays a pivotal role in students’ decisions to either complete or withdraw from online courses [7]. Although benefitting from its most prominent characteristic of no time and space boundaries [69], distance education suffers from the negative impacts it has on transactional distance. Transactional distance, described as the degree of psychological distance between the learner and teacher by Moore [70], needs to be considered to improve learner satisfaction. Another dimension to consider is motivation. Motivated, goal-oriented, and engaged students tend to show better persistence in online learning [52,65,66,71]. Furthermore, previous studies have shown that the decision behind dropout in some cases lies in students’ locus of control [51,66,72,73]. However, there is no single factor on its own that can be held responsible for student dropout. Rather, it is a combination of different factors in different settings that results in non-persistence. Therefore, this brings attention to the significance of instructional design as an aspect of early intervention. According to Chyung et al. [74], an effective online learning environment, where student participation is high, involves well-designed curriculum and course materials that meet expectations and contribute to student satisfaction and motivation, accordingly. When the course is tailored to specific career goals and individual learning styles, as Perry et al. [53] suggest, the students will be less likely to drop out.

In all, the focal point of the aforementioned three themes is that dropout is a multidimensional and multilayered issue that has contextualized traits ranging from the way it is defined, the learning design (learner agency and personalized learning), the learning needs (e.g., formal learning requirements as in the higher education or lifelong learning needs as in the case of MOOCs), and socio-economic background (e.g., time needed, working status, family responsibilities, etc.) along with psychological status (e.g., satisfaction, motivation, engagement, etc.). This implies that the dropout equation has many variables and, to identify these variables and provide possible solution scenarios, future research can approach the research in question from different perspectives.

## 5. Conclusions and Suggestions

The issue of student dropout in distance education systems presents significant challenges for educators, administrators, and policy-making communities. By exploring the research trends and patterns of dropout in distance education systems using data mining and analytics methodologies, this study offers valuable theoretical and practical insights to enhance the current understanding of learner retention in online networked learning environments.

Dropout is a relative term, interpreted differently by different researchers, institutions, and educational systems. In most cases, intervention strategies that work for one system will not necessarily be applicable for another. Likewise, due to the sui generis feature of each online learning system, the reasons given for dropout may be unique to the program. Therefore, in order to avoid misinterpretation and to be able to come up with a proper diagnosis, a precise definition of the term “dropout” in the context of distance education is needed.

Another thing to consider is the excessive emphasis on supervised learning algorithms in building discriminative models to predict dropout. Considering the massive target populations of distance education programs, which include learners from any social, academic, or economic background, prediction models based only on learning algorithms may fall short because of their neglect of unanticipated circumstances of human nature. What is more, these models may lead to the exclusion of learners believed to be prone to dropout and leave them with no choice but failure. Thus, there is a need to develop ethical principles, policies, and frameworks regarding the use of algorithms to predict dropout in distance education. It should also be kept in mind that there may be other psychological and sociological reasons for dropping out of school, and in this context, it should be highlighted that algorithmic solutions cannot identify such reasons.

The findings of this study correlate fairly well with previous works and further support the idea that dropout from distance learning may arise from various factors. What seems to be fundamental to note here is that neither a single factor can be held responsible for student dropout, nor can a single formula ensure persistence. Rather, educators, institutions, and designers of online programs should adopt a human-centered approach that can foster learners’ motivation, satisfaction, and independence. In the end, learning is supposed to be a social process that requires giving more agency to learners and supporting them socially and academically to minimize the dropout rates.

Motivation can be introduced as an umbrella term for many factors, as it can be linked to many other factors to some extent. When learners are more motivated, it can eliminate both environmental- and student-related factors, since the first would better help them balance their family, work, and study life, and the latter would assist them in gaining self-regulation and self-efficacy skills. In order to increase motivation, learners should receive a considerable amount of encouragement, support, user-friendly learning instructions/interface, relevant content, hands-on experience, and guidance. Lastly, one of the most outstanding features of distance education, “learning at one’s own pace”, should be exploited to improve learner independence and autonomy. By doing so, learners can master their time management and workload.

## Figures and Tables

**Table 1:** Search queries used for the inclusion criteria.

Parameters	Search Strings
Subject-specific queries	“Dropout” OR “drop out”
	AND
Field-specific queries	“Distance education” OR “distance teaching” OR “distance learning” OR “remote education” OR “remote learning” OR “remote teaching” OR “online education” OR “online learning” OR “online teaching” OR “online course” OR “elearning” OR “e-learning” OR “m-learning” OR “mlearning” OR “u-learning” OR “ulearning” OR “MOOC*” OR “massive open online course*” OR “educational technology*” OR “open education” OR “open learning” OR “open teaching”

## Data Availability

The datasets used and/or analyzed during the current study are available from the corresponding author on reasonable request.
